# Decreased functional connectivity is associated with increased levels of Cerebral Spinal Fluid soluble-PDGFRβ, a marker of blood brain barrier breakdown, in older adults

**DOI:** 10.1007/s11682-024-00912-8

**Published:** 2024-09-10

**Authors:** Joey A. Contreras, Kimiko Fujisaki, Nancy E. Ortega, Giuseppe Barisano, Abhay Sagare, Ioannis Pappas, Helena Chui, John M. Ringman, Elizabeth B. Joe, Berislav V. Zlokovic, Arthur W. Toga, Judy Pa

**Affiliations:** 1 Department of Neurosciences, Alzheimer’s Disease Cooperative Study (ADCS), University of California, San Diego, CA USA; 2 Mark and Mary Stevens Neuroimaging and Informatics Institute, University of Southern California, Los Angeles, CA USA; 3 Department of Physiology and Neuroscience, Zilkha Neurogenetic Institute, University of Southern California, Los Angeles, CA USA; 4 Department of Neurology, Keck School of Medicine, University of Southern California, Los Angeles, CA USA

**Keywords:** Resting-state functional magnetic resonance imaging (rsfMRI), Default mode network (DMN), BBB breakdown, Soluble platelet-derived growth factor receptor-β (sPDGFRβ), Cognitive impairment

## Abstract

Resting-state functional connectivity (FC) is suggested to be cross-sectionally associated with both vascular burden and Alzheimer's disease (AD) pathology. For instance, studies in pre-clinical AD subjects have shown increases of cerebral spinal fluid soluble platelet-derived growth factor receptor-β (CSF sPDGFRβ, a marker of BBB breakdown) but have not demonstrated if this vascular impairment affects neuronal dysfunction. It’s possible that increased levels of sPDGFRβ in the CSF may correlate with impaired FC in metabolically demanding brain regions (i.e. Default Mode Network, DMN). Our study aimed to investigate the relationship between these two markers in older individuals that were cognitively normal and had cognitive impairment. Eighty-nine older adults without dementia from the University of Southern California were selected from a larger cohort. Region of interest (ROI) to ROI analyses were conducted using DMN seed regions. Linear regression models measured significant associations between BOLD FC strength among seed-target regions and sPDGFRβ values, while covarying for age and sex. Comparison of a composite ROI created by averaging FC values between seed and all target regions among cognitively normal and impaired individuals was also examined. Using CSF sPDGFRβ as a biomarker of BBB breakdown, we report that increased breakdown correlated with decreased functional connectivity in DMN areas, specifically the PCC, and while the hippocampus exhibited an interaction effect using CDR score, this was an exploratory analysis that we feel can lead to further research. Ultimately, we found that BBB breakdown, as measured by CSF sPDGFRβ, is associated with neural networks, and decreased functional connections.

**Supplementary Information:**

The online version contains supplementary material available at 10.1007/s11682-024-00912-8.

## Introduction

Although beta-amyloid and phosphorylated tau are the characteristic neuropathological hallmarks of Alzheimer’s disease (AD), cerebrovascular dysfunction and vascular pathology have been reported to play an important role in the onset and progression of AD. This is the premise of the two-hit vascular hypothesis for AD (Zlokovic, 2011). The first hit proposes an initial insult that damages blood vessels, leading to blood–brain barrier (BBB) dysfunction. The subsequent hit is the resulting diminished brain perfusion, which increases AD pathology in the brain and ultimately leads to neuronal loss.

Patients with AD also show disruptions in functional connectivity, especially within default mode network (DMN) regions (Jones et al., 2011). The DMN is a collection of brain regions that exhibit synchronized low-frequency blood oxygen level-dependent (BOLD) activity, measured using resting-state functional magnetic resonance imaging (rsfMRI). Although these brain regions are spatially segregated, they are intrinsically coactivated and deactivated over time and are considered functionally connected under task-free conditions. The DMN consists of the bilateral parietal lobes, posterior cingulate cortex, medial prefrontal cortex (MPFC), and hippocampi (Raichle et al., 2001).

Decreased functional connectivity among DMN regions has been associated with the neurodegeneration and cognitive decline related to AD (Staffaroni et al., 2018; Hedden et al., 2009; Zhou et al., 2010), possibly due to the DMN’s functional role in autobiographical memory, future thinking, and introspection (Buckner and Carroll, 2007; Buckner et al., 2008).

Although beta-amyloid and phosphorylated tau are the characteristic neuropathological hallmarks of Alzheimer’s disease, cerebrovascular dysfunction and vascular pathology have been reported to serve an important role in the onset and progression of AD. This is the premise of the two-hit vascular hypothesis for AD (Zlokovic, 2011), such that the first hit proposes an initial insult that causes damage to blood vessels leading to BBB dysfunction. Then, the subsequent hit is the resulting diminished brain perfusion which leads to increased AD pathology in the brain and ultimate neuronal loss. Patients with AD also show disruptions in functional connectivity, especially within DMN regions (Jones et al., 2011). The DMN is a collection of brain regions that exhibit synchronized low temporal frequency blood oxygen level dependent (BOLD) activity measured using resting state functional magnetic resonance imaging (rsfMRI). Although the brain regions are spatially segregated, they are intrinsically coactivated and deactivated across time and considered to be functionally connected under task free conditions. The DMN consists of the bilateral parietal lobes, posterior cingulate cortex, medial prefrontal cortex (MPFC), and hippocampi (Raichle et al., 2001). Decreased functional connectivity among DMN regions has been associated with the neurodegeneration and cognitive decline related to AD (Staffaroni et al., 2018; Hedden et al., 2009; Zhou et al., 2010), possibly for the DMN’s functional role in autobiographic memory, future thinking, and introspection (Buckner and Carroll, 2007; Buckner et al., 2008).

The BBB plays a crucial role in regulating the composition of the neuronal internal milieu, which is essential for proper neuronal and synaptic functioning (Sagare et al., 2015; Zhao et al., 2015). Notably, BBB breakdown has been proposed as an early biomarker for Alzheimer's disease (AD), independent of amyloid and tau. For instance, BBB breakdown has been observed in DMN brain regions among individuals with mild cognitive impairment (MCI) (Montagne et al., 2016; Nation et al., 2019; Barisano et al., 2022; Hussain et al., 2021).Using dynamic contrast-enhanced MRI (DCE-MRI), increased BBB permeability was observed in the hippocampi of individuals with very early cognitive impairment compared to age-matched cognitively unimpaired older adults (Montagne et al., 2016). This finding correlated with increased levels of soluble platelet-derived growth factor receptor-β (sPDGFRβ), suggesting that cerebrospinal fluid (CSF) sPDGFRβ may be a biomarker of BBB breakdown via pericyte injury.

Since regions within the DMN appear to function as connectivity 'hubs' and as sites of pathological insults in AD, it is noteworthy that the connections between DMN functional connectivity and BBB breakdown have not been explored despite their relevance to AD risk and pathogenesis. Our study aimed to investigate the correlation between vascular and functional activity changes in individuals with no cognitive impairment and early cognitive impairment. We hypothesized that those with early cognitive impairment would show a significant interaction between sPDGFRβ levels and functional connectivity values. Ultimately, this study evaluated the association between CSF sPDGFRβ and DMN functional connectivity to better characterize the link between these two early indicators of AD risk.

## Methods

### Participants

Participants were recruited through the University of Southern California Alzheimer’s Disease Research Center (ADRC) in Los Angeles, CA. The study and procedures were approved by the Institutional Review Board indicating compliance with all ethical regulations, and informed consent was obtained from all participants prior to study enrollment. All participants underwent neurological and neuropsychological evaluations performed using the Uniform Data Set (UDS) and additional neuropsychological tests, as described below. Eighty-nine, mostly white participants were included based on availability of T1-weighted MPRAGE scan, rsfMRI scan, Clinical Dementia Rating (CDR) score, and CSF sPDGFRβ biomarker data that was collected within 90 days of the MRI scan date. All biomarker assays and quantitative MRI scans were conducted by investigators blinded to the clinical status of the participant.

### Inclusion/exclusion criteria

Individuals were eligible for inclusion if they were aged 45 or above, displayed either normal cognitive function or early cognitive dysfunction, and had no existing or previous neurological or psychiatric disorders that could account for any identified cognitive decline. These disorders included organ failure, brain tumors, epilepsy, hydrocephalus, schizophrenia, major depression, Parkinson’s disease, Lewy body dementia or frontotemporal dementia. Participants could not have current contraindications to MRI and use medications that might explain any observed cognitive impairment.

### Clinical dementia rating (CDR)

Clinical Dementia Rating (CDR) assessments followed the standardized process for CDR interviews. All participants underwent a comprehensive clinical interview, which included their medical history as well as a physical examination. Additionally, individuals with relevant knowledge were also interviewed. For the study's classification, participants with a CDR score of 0 were designated as having no cognitive impairment, while those with a score higher than 0 were classified as experiencing cognitive impairment (Khan, 2016; Mendez, 2022).

### Lumbar puncture and molecular biomarkers in the cerebrospinal fluid (CSF) assays

Participants underwent lumbar puncture in the morning after an overnight fast. CSF was collected in polypropylene tubes, processed (centrifuged at 2000; relative centrifugal force (RCF) for 10 min at 4 °C), aliquoted into polypropylene tubes and stored at − 80 °C until the time of assay.

### APOE genotyping

APOE genotyping was performed as described in Nation et al. (2019). Participants with at least one copy of the E4 allele were considered APOE4 carriers. There were no E4 homozygous carriers.

### Quantitative western blotting of sPDGFRβ

Quantitative Western blot analysis was used to determine CSF levels of sPDGFRβ in human CSF (ng/mL). Standard curves were generated using recombinant human PDGFRβ (Cat. No. 385-PR-100/CF, R&D Systems, Minneapolis, MN) (as described in Nation et al., 2019).

### MRI data acquisition, preprocessing and analysis

All images were obtained on a Siemens TrioTim 3 T scanner using a 20-channel head coil. Anatomical T1-weighted 3D gradient echo pulse sequence scans were acquired with the following parameters: flip angle = 8°, TR/TE = 2400/3.16 ms, FOV 256 × 256 mm, voxel size was 1.1 × 1.1 × 1.2 mm^3^ isotropic, length of scan = 7.04 min. rsfMRI scans were acquired eyes closed with the following parameters: flip angle 90°, TR/TE = 2200/27 ms, FOV 384 × 384 mm, voxel size: 4 mm^3^ isotropic, scan time = 6.01 min.

rsfMRI images were preprocessed using the CONN-toolbox v20a (Whitfield-Gabrieli and Nieto-Castanon, 2012) in SPM12 for data analysis. The preprocessing pipeline of the functional images consisted of motion correction, co-registration to structural images, spatial normalization to the Montreal Neurological Institute (MNI) template, smoothing with a 5 mm full-width at half-maximum Gaussian kernel, and band-pass filtering of 0.009–0.1 Hz. After preprocessing, the CompCor strategy (Behzadi et al., 2007) was implemented to account for white matter and CSF noise using principal component analysis. The analyses did not include global signal regression to avoid potential false anticorrelations (Murphy et al., 2009). Motion parameters, cerebrospinal fluid, and white matter were included in the model and considered as variables of no interest. The mean BOLD signal time course was then extracted from every ROI predefined by the Harvard–Oxford atlas and resting state networks as defined by Shen and colleagues.

(Shen et al., 2013), except for cerebellar and primary sensory areas. The total number of ROIs included in this analysis was, therefore, 138. Pearson’s correlation coefficients were calculated for all pairwise comparisons between ROIs making this an ROI-to-ROI, seed-based analysis.

### Analysis of sPDGFRβ with resting-state functional connectivity using FDR corrected p-value

**Fig. 1:**
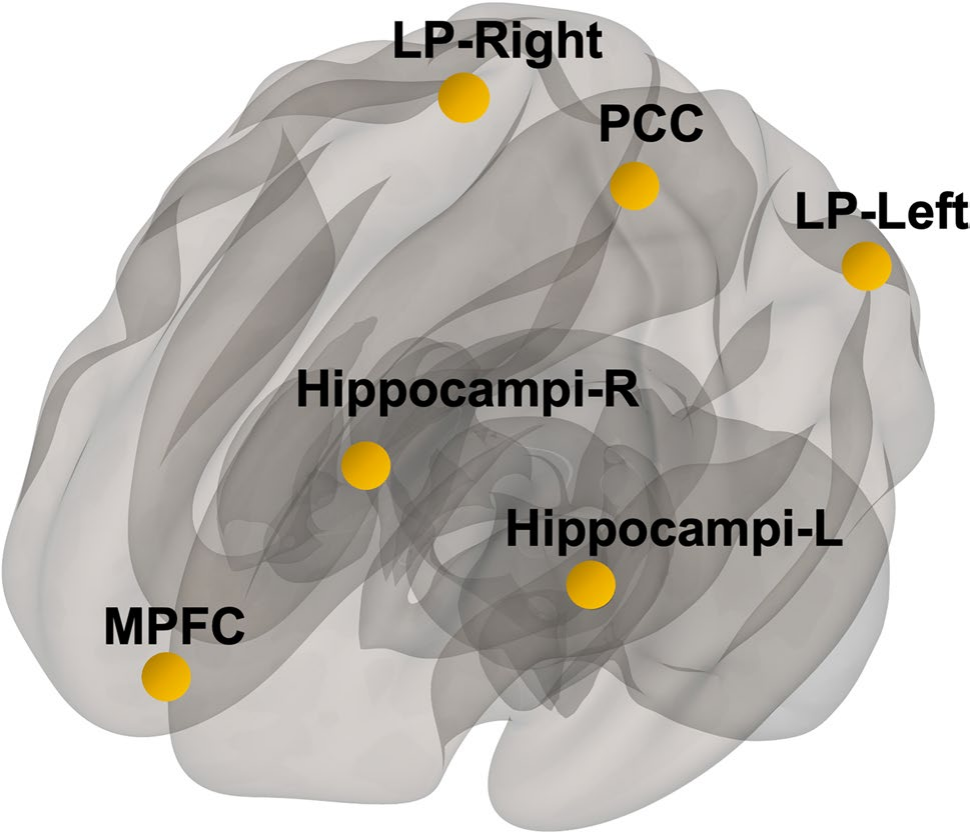
Seed Regions. Default mode network brain regions were selected as seed regions which consisted of medial prefrontal cortex (mPFC), posterior cingulate cortex (PCC), bilateral parietal brain regions (LP-left, LP-right), and bilateral hippocampi regions

Linear regression was used to compute the correlation between all seed and target ROIs functional connectivity (FC) strength and sPDGFRβ. DMN regions of bilateral parietal lobes, posterior cingulate cortex, medial prefrontal cortex, and hippocampi were used as seed regions (Fig. 1). Age and sex were included as model covariates with false discovery rate (FDR) corrected p-value set at 0.05 using the five above mentioned DMN seed regions.

### Exploratory analysis of sPDGFRβ with resting-state functional connectivity using uncorrected p-value of 0.05

Additionally, an uncorrected p-value at 0.05 was also used for an exploratory analysis limiting the number of target ROIs (see supplemental material, Table 1 for complete list of target ROIs used). For these exploratory analyses, a composite ROI was created by averaging the functional connectivity across all DMN seed regions and target regions. This approach revealed other potentially meaningful patterns that could be considered in future analyses.

### Multiple regression analysis using CDR as an interaction term

**Fig. 2:**
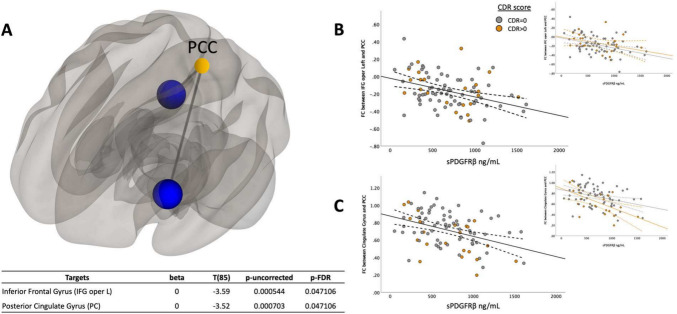
sPDGFRβ Values Negatively Correlated with Resting-State Functional Connectivity using PCC as seed region. Linear regression analysis revealed the functional connectivity between PCC and both the left inferior frontal (R^2^ = 0.13, *t*(85) = -3.59, CI 95% [-2.1 × 10^-4, 6 × 10^-5], p = 0.047) and posterior cingulate gyrus (R^2^ = 0.14, *t*(85) = -3.52, CI 95% -2 × 10^-4, 6 × 10^-5], p = 0.047) was lower as sPDGFRβ values increased displayed on a glass brain shown in Panel **A**. Data were plotted to visualize differences between cognitively normal individuals (colored in grey) versus those with mild cognitive impairment (colored in orange). Panel **B** shows functional connectivity values between PCC and inferior frontal gyrus on the y axis and its relationship to sPDGFRβ values on the x axis. Panel **C** plots functional connectivity between PCC and the posterior cingulate gyrus. Age and sex were used as covariates

**Fig. 3:**
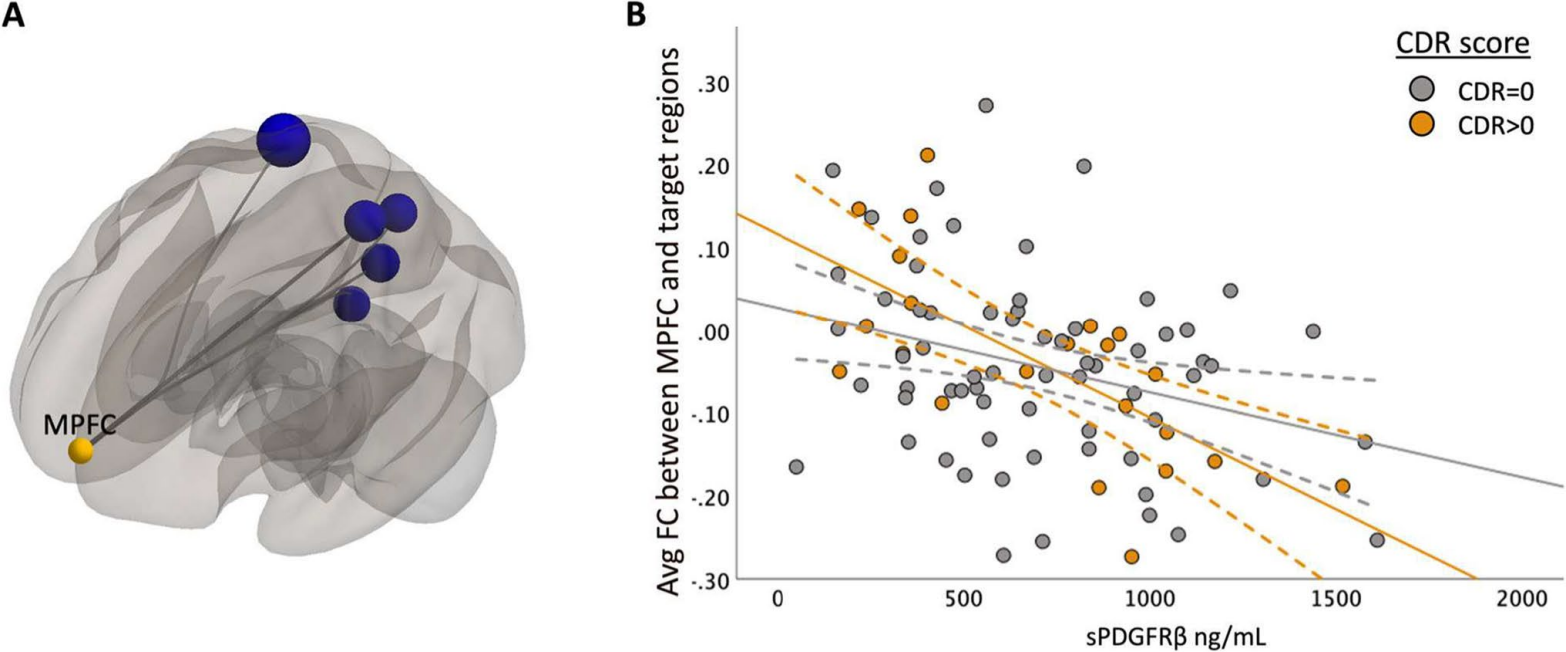
sPDGFRβ Values Negatively Correlated with Resting-State Functional Connectivity using MPFC as seed region with uncorrected p-value. Panel **A** shows decreased FC between DMN seed region medial prefrontal cortex and ROIs within the parietal and frontal lobe are correlated with increased CSF sPDGFRβ values projected on a glass brain. This data is plotted in Panel **B** which uses an avg FC between seed region MPFC and significant target region shown in panel A on the y axis and sPDGFRβ on the x axis. Patients with CDR scores 0.5 and above show a more marked decrease

**Fig. 4:**
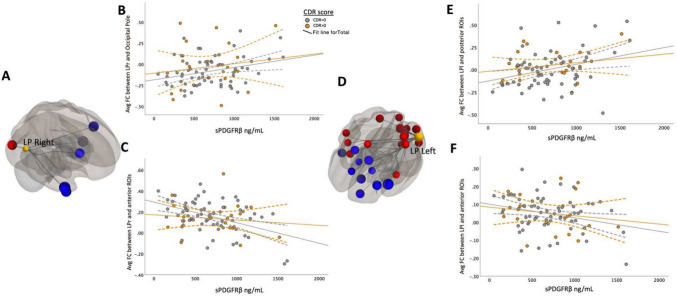
Bilateral parietal seed ROIs revealed both negative and positive correlations between sPDGFRβ values and target ROIs. Panels **A** and **D** show the significant FC between DMN bilateral parietal seed regions that are correlated with increased CSF sPDGFRβ values projected on a glass brain. Significant posterior target brain regions were averaged and plotted in panels **B** and **E** by corresponding sPDGFRβ on the x axis. Plotted data is stratified by color to appreciate how cognitive status affects FC’s relationship with sPDGFRβ (CDR scores above 0.5 coded in orange, CDR scores equaling 0 coded in grey)

**Fig. 5:**
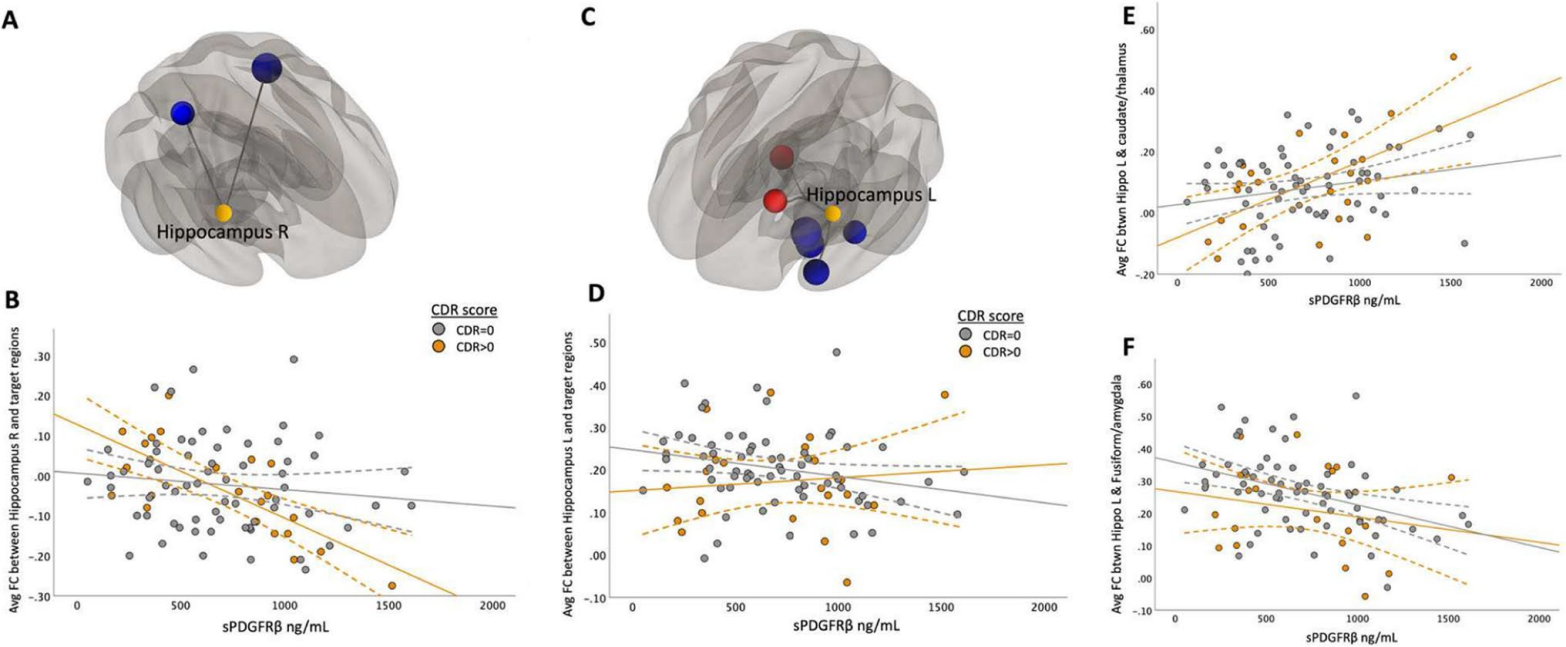
Correlations Between sPDGFRβ Values and Resting-State Functional Connectivity Differed Based on CDR Score when using hippocampus as seed region. The main effect of functional connectivity between right hippocampus and target ROIs in the superior temporal and parietal regions and sPDGFRβ values was significant (*F*(1,84) = 14.64, CI 95% [-2 × 10^-4, 3 × 10^-5], *p* < 0.001, Panel **A**). A significant interaction effect was seen using CDR score as an interaction term showing patients with CDR score higher than zero had greater negative correlation between averaged significant FC and sPDGFRβ values (*F*(1,84) = 6.15, *p* = 0.015, Panel **B**). Left hippocampal seed region showed two clusters that exhibited both positive and negative correlations between FC and sPDGFRβ values (Panel **C**). Panel **D** shows the plots the averaged significant FC values (y-axis) against sPDGFRβ values (x-axis). Panel **E** shows the positive correlation between CSF marker sPDGFRβ and FC values between the left hippocampus and caudate and thalamus (*F*(1,84) = 13.16, CI 95% [3 × 106–4, 2 × 106–5], *p* < 0.001) showing a significant interaction with CDR score. Panel **F** shows the negative correlation between sPDGFRβ and FC consisted of target regions in the fusiform gyrus, parahippocampus and amygdala region (Fig. 5F, Table 2)

To understand how cognitive status (cognitively unimpaired vs. cognitively impaired) affected the relationship between sPDGFRβ values and resting-state functional connectivity, all data were plotted to visualize differences between cognitively normal individuals (colored in grey) versus those with mild cognitive impairment (colored in orange), Figs. 2, 3, 4 and 5 and an interaction analysis was done to determine if there was a difference in the relationship between sPDGFRβ (y variable) with Resting-State Functional Connectivity (x-variable) and using CDR as the interaction term.

## Results

### Demographic characteristics of the cohort

A total of 89 participants (67 cognitively unimpaired, 22 cognitively impaired individuals) were included in this study. Of the total sample, 41 participants were male and 41 were *APOE*4 carriers. Between cognitively unimpaired and impaired individuals, there was no difference in age, or CSF marker sPDGFRβ values. Results are summarized in Table 1.

**Table 1:** Participant Demographics. Cohort characteristics used in analysis. Race information was provided for 80 participants (9 participants did not have recorded data). Significance was set at p-value < 0.05

	Total Sample (N = 89)	CDR = 0 (N = 67)	CDR > 0 (N = 22)	P-Value
MRI age, mean(s.d.)	64.83 (11.73)	64.57 (10.43)	65.5 (15.43)	0.68
Sex (m/f)	41/48	31/36	11-Nov	0.64
Race	80	58	22	–
White	66	49	17	
African-American	1	1	0	
Native American	4	2	2	
Eskimo	1	0	1	
Aluet	8	6	2	
Missing data	9	9	0	
APOE4 status (noncarrier/carrier)	48/41	37/30	11-Nov	0.73
CDR (0/0.5/1/3)	67/18/3/1	n/a	n/a	–
sPDGFRβ (ng/mL), mean(s.d.)	685.63 (351.72)	695.99 (347.30)	704.96 (369.54)	0.92
Averaged FC among DMN regions, mean (s.d.)	0.06 (0.07)	0.06 (0.07)	0.05(0.07)	0.86

### sPDGFRβ values negatively correlated with resting-state functional connectivity using PCC as seed region

Using the DMN seed region posterior cingulate cortex (PCC), a significant negative correlation was found between sPDGFRβ and functional connectivity values. Specifically, the functional connectivity between PCC and both the left inferior frontal (R^2^ = 0.13, *t*(85) = -3.59, CI 95% [-2.1 × 10^-4, 6 × 10^-5], p = 0.047) and posterior cingulate gyrus (R^2^ = 0.135, *t*(85) = -3.52, CI 95% -2 × 10^-4, 6 × 10^-5], p = 0.047) was lower as sPDGFRβ values increased (Fig. 2).

### Exploratory analysis using uncorrected p-value of 0.05

When using the MPFC as a seed region, there was a significant negative correlation between higher sPDGFRβ values and functional connectivity values between MPFC and 6 ROIs (Fig. 3) which include the precuneus, right cuneal cortex, posterior cingulate cortex (PCC), right lingual gyrus (rLG), right superior parietal lobule (rSPL), and right intracalcarine cortex (rICC).

Bilateral parietal seed ROIs revealed both negative and positive correlations between sPDGFRβ values and seed and target ROIs (listed in Table 2).

**Table 2:** Significant Target Regions using DMN brain regions as Seeds

DMN Seed Region 1: Medial Prefrontal Cortex (MPFC)		
**Targets**	**T(85)**	**p-value**
SPL r (Superior Parietal Right)	-3.13	0.002
Precuneous (Precuneous Cortex)	-2.5	0.014
ICC r (Intracalcarine Cortex right)	-2.32	0.023
Cuneal r (Cuneal Cortex Right)	-2.31	0.023
LG r (Lingual Gyrus Right)	-2.2	0.031
DefaultMode RSN: Posterior Cingulate Cortex (PCC)	-2.07	0.041
DMN Seed Region 2: Posterior Cingulate Cortex (PCC)		
Targets	**T(85)**	**p-value**
IFG oper l (Inferior Frontal Left)	**-3.59**	**0.001**
PC (Posterior Cingulate Gyrus)	**-3.52**	**0.001**
FO l (Frontal Operculum Left)	-2.78	0.007
Caudate r	-2.73	0.008
aMTG r (Middle Temporal Gyrus Right)	-2.6	0.011
SFG r (Superior Frontal Gyrus Right)	-2.54	0.013
Caudate l	-2.48	0.015
PaCiG r (Paracingulate Gyrus Right)	-2.3	0.024
Accumbens r	-2.27	0.026
aITG r (Inferior Temporal Right)	-2.2	0.031
PaCiG l (Paracingulate Gyrus Left)	-2.17	0.033
pMTG l (Middle Temporal Gyrus Left)	-2.15	0.034
Language RSN: pSTG (L)	-2.12	0.037
DefaultMode RSN: Medial prefrontal cortex (MPFC)	-2.07	0.041
AG l (Angular Gyrus Left)	-2.07	0.042
aITG l (Inferior Temporal Left)	-2.05	0.044
toMTG r (Middle Temporal Right)	-2.04	0.045
DMN Seed Region 3: Hippocampus (R)		
Targets	**T(85)**	**p-value**
Language RSN: pSTG (L)	-2.48	0.015
SPL r (Superior Parietal Lobe)	-2	0.049
Hippocampus (L)		
Amygdala l	-2.64	0.010
aPaHC l (anterior Parahippocampal Cortex)	-2.41	0.018
aTFusC l (anterior Temporal Fusiform)	-2.23	0.029
Thalamus r	2.11	0.038
Caudate l	2.04	0.045
pTFusC l (posterior Temporal Fusiform)	-2.01	0.047
DMN Seed Region 4: Lateral Parietal Right (LP)		
Targets	**T(85)**	**p-value**
aMTG r (Middle Temporal Gyrus Right)	-2.51	0.014
aMTG l (Middle Temporal Gyrus Left)	-2.35	0.021
IFG oper l (Inferior Frontal Left)	-2.29	0.025
aITG r (Inferior Temporal Right)	-2.28	0.025
Caudate r	-2.17	0.033
OP r (Occipital Pole Right)	2.14	0.035
Lateral Parietal Left (LP)		
IFG oper l (Inferior Frontal Left)	-2.96	0.004
FrontoParietal RSN: Lateral Prefrontal Cortex (LPFC)	-2.59	0.011
SubCalC (Subcallosal Cortex)	-2.56	0.012
SFG l (Superior Frontal G*eft)	-2.51	0.014
ICC l (Intracalcarine Cortex Left)	2.49	0.015
aMTG r (Middle Temporal Gyrus right)	-2.45	0.016
FP l (Frontal Pole Left)	-2.45	0.016
Visual RSN: Medial	2.41	0.018
SFG r (Superior Frontal Gyrus right)	-2.38	0.019
OP l (Occipital Pole Left)	2.32	0.023
DorsalAttention RSN: IPS	2.3	0.024
Visual RSN: BiLateral Regions	2.27	0.030
IC r (Insular Cortex Right)	2.16	0.034
SPL l (Superior Parietal Left)	2.15	0.034
OP r (Occipital Pole Right)	2.13	0.036
LG l (Lingual Gyrus Left)	2.12	0.037
PaCiG r (Paracingulate Gyrus Right)	-2.12	0.037
SensoriMotor RSN: Lateral Region	2.1	0.039
Sensory Motor Area R (SMA)	2.08	0.041
Caudate l	-2.07	0.041
CO r (Central Opercular Right)	2.02	0.046
Visual.Occipital	2.02	0.047
CO l (Central Opercular Left)	2.02	0.047
Caudate r	-1.99	0.049

When brain regions were divided via an anterior–posterior axis, different relationships with sPDGFRβ values were observed. FC between parietal seed regions and anterior regions correlated negatively to increased BBB breakdown whereas FC between parietal seed regions and posterior regions correlated positively to BBB breakdown (shown in Fig. 4, Table 2).

The correlation between functional connectivity and sPDGFRβ in the right hippocampus and target ROIs in the superior temporal and parietal regions was significant (*F*(1,84) = 14.64, CI 95% [-2 × 10^-4, 3 × 10^-5], *p* < 0.001, Fig. 5A-B). The left hippocampal seed region showed two clusters that exhibited both positive and negative correlations between FC and sPDGFRβ values. Specifically, there was found to be a positive correlation between CSF marker sPDGFRβ and FC values between the left hippocampus, caudate and thalamus (*F*(1,84) = 13.16, CI 95% [3 × 106–4, 2 × 106–5], *p* < 0.001). The negative correlation between sPDGFRβ and FC consisted of target regions in the fusiform gyrus, parahippocampus, and amygdala region (Fig. 5F, Table 2).

### Correlations between sPDGFRβ values and resting-state functional connectivity by CDR score

When using seed regions PCC (Fig. 2C) and mPFC (Fig. 3B), there was no significant interaction using CDR score. The bilateral hippocampi regions exhibited an interaction effect. A significant interaction effect was observed for functional connectivity between right hippocampus and temporal/parietal regions and sPDGFRβ values when using CDR score as an interaction term, such that cognitively impaired patients (CDR > 0) had greater negative correlation between FC and sPDGFRβ values (*F*(1,84) = 6.15, *p* = 0.015, Fig. 5B). The left hippocampus had a significant interaction with CDR score such that a CDR score higher than zero had a greater positive correlation between functional connectivity between left hippocampus and thalamus and caudate and sPDGFRβ (*F*(1,84) = 13.16, *p* = 0.045, Fig. 5C,E). When the bilateral parietal regions were used as seed ROIs, no interaction effects were observed with CDR score.

**Fig. 6:**
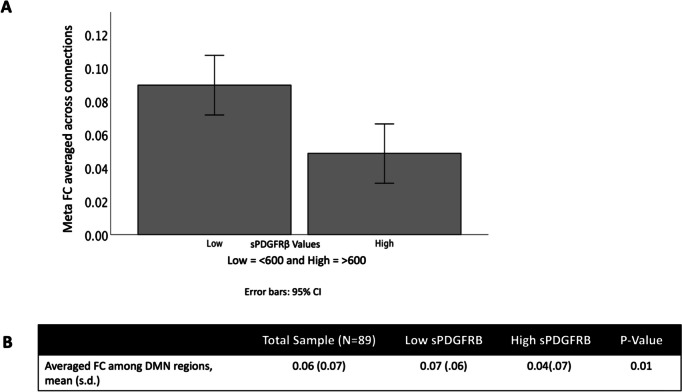
sPDGFRβ Values Negatively Correlated with Resting-State Functional Connectivity overall. An overall decrease in functional connectivity using DMN seed regions and significant target regions was found when sPDGFRβ values were high (*t*(88) = 6.96, *p* = 0.01)

Across all participants, we found an overall decrease in functional connectivity using a composite ROI (which combined the functional connectivity across all seeds and targets) when sPDGFRβ values were high (*t*(88) = 6.96, 95% CI [4 × 10^-3, 0.20], *p* = 0.01, Fig. 6).

## Discussion

Previous studies have shown that the loss of structural integrity of the blood–brain barrier (BBB) correlates with early cognitive dysfunction (Nation et al., 2019; Sengillo et al., 2013; Montagne et al., 2020), independent of amyloid-beta (Aβ) and tau PET levels. However, it is unknown whether this structural breakdown of the BBB is reflected in resting-state functional connectivity. Resting-state MRI can measure functional connectivity changes in the early stages of AD, particularly in brain areas known to be affected, such as the DMN.

To gain a more complete understanding of the pathogenic role of BBB breakdown in the brain, we felt it important to investigate the relationship between vascular and functional activity changes in brain regions known to be adversely affected in both cognitively unimpaired individuals and those with very early cognitive impairment. While we did not find a significant interaction between individuals with early cognitive impairment and those with no cognitive impairment, we did report that increased BBB breakdown correlated with decreased functional connectivity in DMN areas, specifically the posterior cingulate cortex (PCC), when using cerebrospinal fluid (CSF) soluble platelet-derived growth factor receptor-β (sPDGFRβ) as a biomarker of BBB breakdown.

The PCC is an important region of the traditional DMN and plays key roles in episodic memory, spatial attention, self-evaluation, and other cognitive functions (Braak & Braak, 1991; Greicius et al., 2003; Gusnard et al., 2001; Ries et al., 2006). Numerous studies have found diminished functional connectivity between the PCC and the brain neocortex in patients with early AD and those carrying AD-susceptible genes, suggesting reduced connectivity between the PCC and the medial temporal lobe, where initial histopathological changes occur in AD (Braak & Braak, 1991). It is believed that injury to the medial temporal lobe directly affects its functional connectivity with the PCC, leading to decreased metabolic activity within the PCC (Wang et al., 2009). Additionally, there is a widespread loss of connectivity within the neocortex.

Zhong et al. (2014) found that the PCC serves as a convergence center that receives interactions from most other regions in the DMN. It has been speculated that the PCC integrates signals within the DMN and plays a role in memory identification, storage, and extraction (Miao et al., 2011). Additionally, Esposito et al. (2013) found that outside of the DMN, functional connectivity between the PCC and the left frontal gyrus was weakened in mild cognitive impairment (MCI) patients. This is consistent with our findings, which show lower functional connectivity between the PCC and the frontal and cingulate gyri in participants with more BBB breakdown, as measured by CSF sPDGFRβ.

Using dynamic contrast-enhanced MRI (DCE-MRI) with gadolinium-based contrast agents, it was revealed that BBB breakdown occurs early in individuals with MCI and AD-type dementia, as indicated by the presence of gadolinium (an indicator of subtle BBB leaks) in the brain (Montagne et al., 2015; Nation et al., 2019). With the finding that lower functional connectivity is associated with higher CSF sPDGFRβ, we further conclude that BBB permeability is associated with resting-state functional connectivity, suggesting a link between BBB leakage and neural pathology in AD.

To expand this knowledge, we explored whether other DMN regions showed similar results. Examining uncorrected significant correlations between functional connectivity and CSF sPDGFRβ, we found many consistent results showing decreased functional connectivity between DMN regions correlating with increased BBB breakdown. Notably, bilateral parietal seed ROIs revealed both negative and positive correlations with sPDGFRβ values. These relationships were distinctly different along an anterior–posterior division, such that functional connectivity between the lateral parietal and anterior regions correlated negatively with increased BBB breakdown, while posterior regions correlated positively with BBB breakdown. This anterior–posterior division supports a disconnection syndrome due to alterations in structural and functional integrity (Delbeuck et al., 2003). The disconnection in long-distance functional connectivity between DMN hubs and the anterior brain area, significantly correlated with cognitive impairment (Liu et al., 2014), is another characteristic of AD (Zhang et al., 2009). One study found that cognitively impaired individuals demonstrated decreased functional connectivity in the posterior region of the retrosplenial cortex and the more anterior prefrontal cortex (Yasuno et al., 2015). Similar to our findings, Tao et al. (2020) found a transitional stage of functional connectivity in AD progression that presents as a disconnection between anterior and posterior brain regions among individuals in the earliest stages of cognitive decline. It is possible that we are observing an early consequence of pathology that results in a distinct disconnect between how the anterior and posterior portions of the brain communicate with each other. Ultimately, although results are inconsistent, we hope to continue adding knowledge to this field to understand the neurological mechanisms underlying cognitive decline, BBB breakdown, and functional connectivity in the context of AD.

To gain a clearer understanding of whether cognitive impairment drives the relationship between BBB breakdown and functional connectivity, all data were plotted to visualize differences between cognitively normal individuals (colored in grey) and those with mild cognitive impairment (colored in orange). Interaction effects were tested and observed only in the hippocampus. Using the Clinical Dementia Rating (CDR) score as an interaction term, we found that the relationship between sPDGFRβ and functional connectivity between the right hippocampus and target ROIs in the superior temporal and parietal regions differed significantly compared to individuals with a CDR score of 0. This was also the case when examining the relationship between sPDGFRβ and the left hippocampus, caudate, and thalamus regions. We had expected to find greater effects among our cognitively impaired group across multiple DMN brain regions. We hypothesize that increased sPDGFRβ levels may affect functional connectivity first, making this marker more sensitive to neuronal disruption before it is present in cognitively impaired individuals, as also discussed in Nation et al., 2019. However, the fact that we observed effects only in the hippocampus is consistent with previous work showing that BBB permeability is specifically increased in the hippocampus. Montagne and colleagues also reported that BBB breakdown during normal aging and MCI starts in the hippocampus (Montagne et al., 2015). Our results add to this knowledge by incorporating functional connectivity and indicate that these measures are sensitive to cognitive impairment.

The main limitation of the present study is the smaller sample size, which was not adequately powered to stratify results by CDR and APOE4 carriers versus non-carriers, as well as an unequal sample of those with a CDR score greater than zero. This limitation was primarily due to the requirement of matching MRI, CSF, and CDR data within 90 days of each other. For this reason, we wanted to expand our results to be more exploratory, directing future research but recognizing that this approach increases the risk of type I error. Additionally, due to the cross-sectional nature of this study, causality in these relationships cannot be assumed. Given that this was not longitudinal data, it cannot be assumed that all individuals are on an AD trajectory, making this population potentially heterogeneous in disease/pathology. Lastly, we acknowledge that obtaining a representative and diverse population for this study was not prioritized during recruitment, and we therefore cannot determine if race played a significant role in the relationship between BBB breakdown and functional connectivity.

## Conclusions

We conclude that BBB breakdown, as measured by CSF sPDGFRβ, is associated with neural networks and decreased functional connectivity, independent of cognitive impairment. This observation suggests a potential relationship between BBB breakdown and functional connectivity, possibly resulting from disruptions in cerebral blood flow as shown in previous literature. This may serve as an earlier indicator of brain degeneration, preceding cognitive impairment.

## Supplementary Information

Below is the link to the electronic supplementary material.

Supplementary file1 (DOCX 208 KB)

## Data Availability

All imaging and demographic data are available in the Image and Data Archive (IDA) at Loni: https://ida.loni.usc.edu. All other data are available upon request from corresponding author.

## References

[CR1] Barisano, G., Montagne, A., Kisler, K., Schneider, J. A., Wardlaw, J. M., & Zlokovic, B. V. (2022). Blood-brain barrier link to human cognitive impairment and Alzheimer’s Disease. *Nature Cardiovascular Research,**1*(2), 108–115. 10.1038/s44161-021-00014-4 35450117 10.1038/s44161-021-00014-4 PMC9017393

[CR2] Behzadi, Y., Restom, K., Liau, J., & Liu, T. T. (2007). A component based noise correction method (CompCor) for BOLD and perfusion based fMRI. *Neuroimage, 37*(1), 90–101. 10.1016/j.neuroimage.2007.04.042 10.1016/j.neuroimage.2007.04.042 PMC2214855 17560126

[CR3] Braak, H., & Braak, E. (1991). Neuropathological stageing of Alzheimer- related changes. *Acta Neuropathologica,**82*(4), 239–259. 1759558 10.1007/BF00308809

[CR4] Buckner, R. L., & Carroll, D. C. (2007). Self-projection and the brain. *Trends in Cognitive Sciences,**11*(2), 49–57. 10.1016/j.tics.2006.11.004 17188554 10.1016/j.tics.2006.11.004

[CR5] Buckner, R. L., Andrews-Hanna, J. R., & Schacter, D. L. (2008). The Brain’s Default Network. *Annals of the New York Academy of Sciences,**1124*, 138. 10.1196/annals.1440.011 10.1196/annals.1440.011 18400922

[CR6] Delbeuck, X., Van der Linden, M., & Collette, F. (2003). Alzheimer’s disease as a disconnection syndrome? *Neuropsychology Review,**13*(2), 79–92. 10.1023/A:1023832305702 12887040 10.1023/a:1023832305702

[CR7] Esposito, R., Mosca, A., Pieramico, V., Cieri, F., Cera, N., & Sensi, S. L. (2013). Characterization of resting state activity in MCI individuals. *PeerJ,**1*, e135. 24010015 10.7717/peerj.135 PMC3757508

[CR8] Greicius, M. D., Krasnow, B., Reiss, A. L., & Menon, V. (2003). Functional connectivity in the resting brain: A network analysis of the default mode hypothesis. *Proceedings of the National Academy of Sciences of the United States of America,**100*(1), 253–258. 12506194 10.1073/pnas.0135058100 PMC140943

[CR9] Gusnard, D. A., Raichle, M. E., & Raichle, M. E. (2001). Searching for a baseline: Functional imaging and the resting human brain. *Nature Reviews Neuroscience,**2*(10), 685–694. 11584306 10.1038/35094500

[CR10] Hedden, T., Van Dijk, K. R., Becker, J. A., Mehta, A., Sperling, R. A., Johnson, K. A., & Buckner, R. L. (2009). Disruption of functional connectivity in clinically normal older adults harboring amyloid burden. *Journal of Neuroscience,**29*(40), 12686–12694. 10.1523/JNEUROSCI.3189-09.2009 19812343 10.1523/JNEUROSCI.3189-09.2009 PMC2808119

[CR11] Hussain, B., Fang, C., & Chang, J. (2021). Blood–brain barrier breakdown: An emerging biomarker of cognitive impairment in normal aging and dementia. *Frontiers in Neuroscience,**15*, 688090. 34489623 10.3389/fnins.2021.688090 PMC8418300

[CR12] Jones, D. T., Machulda, M. M., Vemuri, P., McDade, E. M., Zeng, G., Senjem, M. L., Gunter, J. L., Przybelski, S. A., Avula, R. T., Knopman, D. S., Boeve, B. F., Petersen, R. C., & Jack, C. R., Jr. (2011). Age-related changes in the default mode network are more advanced in Alzheimer disease. *Neurology,**77*(16), 1524–1531. 10.1212/WNL.0b013e318233b33d 21975202 10.1212/WNL.0b013e318233b33d PMC3198977

[CR13] Khan, T. K. (2016). Chapter 2 - Clinical diagnosis of Alzheimer’s disease. In T. K. Khan (Ed.), *Biomarkers in Alzheimer’s Disease* (pp. 27–48). Academic Press. 10.1016/B978-0-12-804832-0.00002-X

[CR14] Liu, Y., Yu, C., Zhang, X., et al. (2014). Impaired long distance functional connectivity and weighted network architecture in Alzheimer’s disease. *Cerebral Cortex,**24*(6), 1422–1435. 23314940 10.1093/cercor/bhs410 PMC4215108

[CR15] Mendez, M. (2022). Chapter 16 - General mental status scales, rating instruments, and behavior inventories. In M. Mendez (Ed.), *The mental status examination handbook* (pp. 181–199). Elsevier. 10.1016/B978-0-323-69489-6.00016-4

[CR16] Miao, X., Wu, X., Li, R., Chen, K., & Yao, L. (2011). Altered connectivity pattern of hubs in default-mode network with Alzheimer’s disease: An Granger causality modeling approach. *PLoS ONE,**6*(10), e25546. 22022410 10.1371/journal.pone.0025546 PMC3191142

[CR17] Montagne, A., et al. (2015). Blood-brain barrier breakdown in the aging human hippocampus. *Neuron,**85*, 296–302. [PubMed: 25611508]. 25611508 10.1016/j.neuron.2014.12.032 PMC4350773

[CR18] Montagne, A., Nation, D. A., Pa, J., Sweeney, M. D., Toga, A. W., & Zlokovic, B. V. (2016). Brain imaging of neurovascular dysfunction in Alzheimer’s disease. *Acta Neuropathologica,**131*(5), 687–707. 10.1007/s00401-016-1570-0 27038189 10.1007/s00401-016-1570-0 PMC5283382

[CR19] Montagne, A., Nation, D. A., Sagare, A. P., Barisano, G., Sweeney, M. D., Chakhoyan, A., Pachicano, M., Joe, E., Nelson, A. R., D’Orazio, L. M., Buennagel, D. P., Harrington, M. G., Benzinger, T. L. S., Fagan, A. M., Ringman, J. M., Schneider, L. S., Morris, J. C., Reiman, E. M., Caselli, R. J., … Zlokovic, B. V. (2020). APOE4 leads to blood-brain barrier dysfunction predicting cognitive decline. *Nature,**581*(7806), 71–76. 10.1038/s41586-020-2247-3 32376954 10.1038/s41586-020-2247-3 PMC7250000

[CR20] Murphy, K., Birn, R. M., Handwerker, D. A., Jones, T. B., & Bandettini, P. A. (2009). The impact of global signal regression on resting state correlations: Are anti-correlated networks introduced? *Neuroimage, 44*(3), 893–905. 10.1016/j.neuroimage.2008.09.036 10.1016/j.neuroimage.2008.09.036 PMC2750906 18976716

[CR21] Nation, D. A., et al. (2019). Blood–brain barrier breakdown is an early biomarker of human cognitive dysfunction. *Nature Medicine,**25*, 270–276. 30643288 10.1038/s41591-018-0297-y PMC6367058

[CR22] Raichle, M. E., MacLeod, A. M., Snyder, A. Z., Powers, W. J., Gusnard, D. A., & Shulman, G. L. (2001). A default mode of brain function. *Proceedings of the National Academy of Sciences of the United States of America,**98*, 676–682. 10.1073/pnas.98.2.676 11209064 10.1073/pnas.98.2.676 PMC14647

[CR23] Ries, M. L., Schmitz, T. W., Kawahara, T. N., Torgerson, B. M., Trivedi, M. A., & Johnson, S. C. (2006). Task-dependent posterior cingulate activation in mild cognitive impairment. *NeuroImage,**29*(2), 485–492. 16102979 10.1016/j.neuroimage.2005.07.030 PMC2627779

[CR24] Sagare, A. P., Sweeney, M. D., Makshanoff, J., & Zlokovic, B. V. (2015). Shedding of soluble platelet-derived growth factor receptor-β from human brain pericytes. *Neuroscience Letters,**607*, 97–101. 10.1016/j.neulet.2015.09.025 26407747 10.1016/j.neulet.2015.09.025 PMC4631673

[CR25] Sengillo, J. D., Winkler, E. A., Walker, C. T., Sullivan, J. S., Johnson, M., & Zlokovic, B. V. (2013). Deficiency in mural vascular cells coincides with blood-brain barrier disruption in Alzheimer’s disease. *Brain Pathology,**23*(3), 303–310. 10.1111/bpa.12004 23126372 10.1111/bpa.12004 PMC3628957

[CR26] Shen, X., Tokoglu, F., Papademetris, X., & Constable, R. T. (2013). Groupwise whole-brain parcellation from resting-state fMRI data for network node identification. *Neuroimage, 82*, 403–415. 10.1016/j.neuroimage.2013.05.081 10.1016/j.neuroimage.2013.05.081 PMC3759540 23747961

[CR27] Staffaroni, A. M., Brown, J. A., Casaletto, K. B., Elahi, F. M., Deng, J., Neuhaus, J., Cobigo, Y., Mumford, P. S., Walters, S., Saloner, R., Karydas, A., Coppola, G., Rosen, H. J., Miller, B. L., Seeley, W. W., & Kramer, J. H. (2018). The longitudinal trajectory of default mode network connectivity in healthy older adults varies as a function of age and is associated with changes in episodic memory and processing speed. *Journal of Neuroscience,**38*(11), 2809–2817. 10.1523/JNEUROSCI.3067-17.2018 29440553 10.1523/JNEUROSCI.3067-17.2018 PMC5852659

[CR28] Tao, W., Sun, J., Li, X., Shao, W., Pei, J., Yang, C., Wang, W., Xu, K., Wang, J., & Zhang, Z. (2020). The Anterior-posterior Functional Connectivity Disconnection in the Elderly with Subjective Memory Impairment and Amnestic Mild Cognitive Impairment. *Current Alzheimer Research,**17*(4), 373–381. 10.2174/1567205017666200525015017 32448103 10.2174/1567205017666200525015017

[CR29] Wang, H., Golob, E., Bert, A., Nie, K., Chu, Y., Dick, M. B., et al. (2009). Alterations in regional brain volume and individual MRI-guided perfusion in normal control, stable mild cognitive impairment, and MCI-AD converter. *Journal of Geriatric Psychiatry and Neurology,**22*(1), 35–45. 19150973 10.1177/0891988708328212

[CR30] Whitfield-Gabrieli, S., & Nieto-Castanon, A. (2012). CONN: A functional connectivity toolbox for correlated and anticorrelated brain networks. *Brain Connectivity, 2*(3), 125–141. 10.1089/brain.2012.0073 22642651

[CR31] Yasuno, F., Kazui, H., Yamamoto, A., et al. (2015). Resting-state synchrony between the retrosplenial cortex and anterior medial cortical structures relates to memory complaints in subjective cognitive impairment. *Neurobiology of Aging,**36*(6), 2145–2152. 25862421 10.1016/j.neurobiolaging.2015.03.006

[CR32] Zhang, H.-Y., Wang, S.-J., Xing, J., et al. (2009). Detection of PCC functional connectivity characteristics in resting-state fMRI in mild Alzheimer’s disease. *Behavioural Brain Research,**197*(1), 103–108. 10.1016/j.bbr.2008.08.012 18786570 10.1016/j.bbr.2008.08.012

[CR33] Zhao, Z., Nelson, A. R., Betsholtz, C., & Zlokovic, B. V. (2015). Establishment and dysfunction of the blood-brain barrier. *Cell,**163*(5), 1064–1078. 26590417 10.1016/j.cell.2015.10.067 PMC4655822

[CR34] Zhong, Y., Huang, L., Cai, S., Zhang, Y., von Deneen, K. M., Ren, A., et al. (2014). Altered effective connectivity patterns of the default mode network in Alzheimer’s disease: An fMRI study. *Neuroscience Letters,**578*, 171–175. 24996191 10.1016/j.neulet.2014.06.043 PMC6293460

[CR35] Zhou, J., Michael, D., Greicius, E. D., Gennatas, M. E., Growdon, J. Y., Jang, G. D., Rabinovici, J. H., Kramer, M. W., Miller, B. L., & Seeley, W. W. (2010). Divergent network connectivity changes in behavioural variant frontotemporal dementia and Alzheimer’s disease. *Brain,**133*(5), 1352–1367. 10.1093/brain/awq075 20410145 10.1093/brain/awq075 PMC2912696

[CR36] Zlokovic, B. V. (2011). Neurovascular pathways to neurodegeneration in Alzheimer’s disease and other disorders. *Nature Reviews Neuroscience,**12*(12), 723–738. 10.1038/nrn3114 22048062 10.1038/nrn3114 PMC4036520

