# Supplementary material for: Decreased functional connectivity is associated with increased levels of Cerebral Spinal Fluid soluble-PDGFRβ, a marker of blood brain barrier breakdown, in older adults
Source: Brain Imaging Behav. Author manuscript; Available in PMC 2025 Jan 6. (PMC11680618; doi:10.1007/s11682-024-00912-8)
Supplement: Supplemental Material [file NIHMS2031506-supplement-Supplemental_Material.docx]

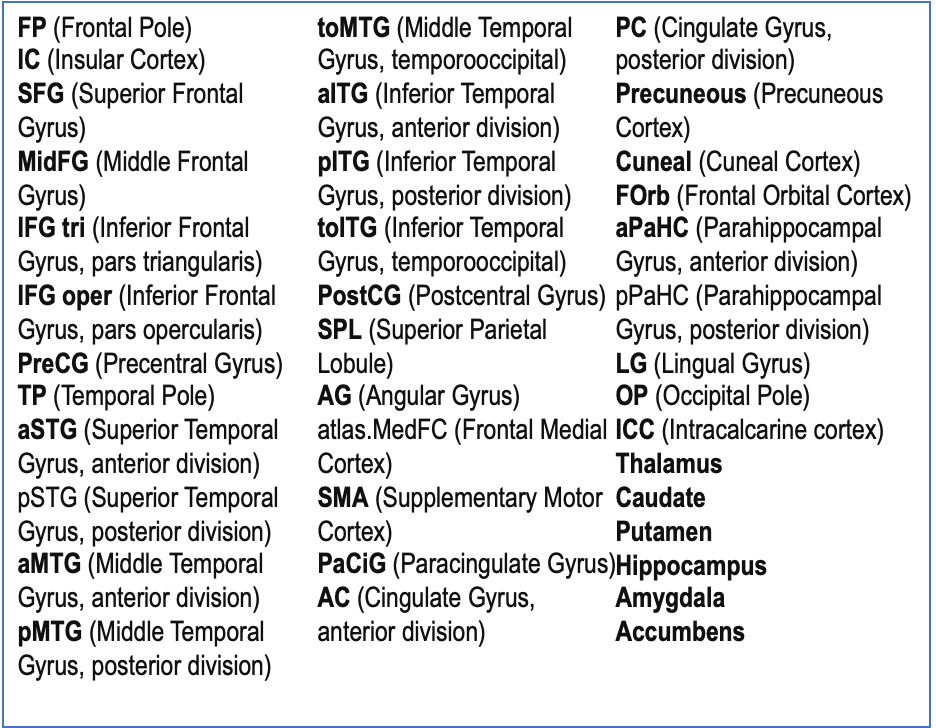


Supplementary Table 1: **Target Regions** were selected to exclude cerebellar and other brain stem regions and include regions that play a role in both executive function and memory largely in the frontal and middle temporal lobe areas.
